# Seasonable Appeals in 1914

**Published:** 1914-12-05

**Authors:** 


### SEASONABLE APPEALS IN 1914.

WE think it may interest a good many of our readers to publish a few actual appeals issued at this season in an up-to-date dress.

The Royal Victoria Infirmary, Newcastle-on-Tyne, has a Subscriptions Committee, consisting largely of working men. It was reorganised in 1905. In the subsequent years, less than nine, its annual income has increased by £17,000. Each month a report is prepared showing the position of the income to date under all heads, and a copy of it is sent to every member of the Subscriptions Committee. In this way each member is seized with knowledge, and contributes by personal efforts to the beating of each previous record. The Committee's special appeal and continuous effort since the war began have prevented the income in 1914 from falling off, and have increased the gross income to date as compared with 1913. *Apropos* of Christmas the Subscriptions Committee's final year's end effort rests on a reprint of the article from THE HOSPITAL of October 10, 1914, entitled "What a Storehouse is the Voluntary Hospital!" Up-to-date appealing at Newcastle takes the form of system and direct personal effort. A welcome and striking feature of up-to-date appeals is their brevity. Here is one issued by

THE POPLAR HOSPITAL FOR ACCIDENTS.

EAST INDIA DOCK ROAD, POPLAR, E.

DEAR SIR,— December 1914.

The War has compelled us to abandon our Annual Dinner, at which we usually obtain about £4,000. This sum is one-third of the income required to maintain the Hospital and the Convalescent Home at Walton-on-the-Naze.

Serious accidents and other casualties—**just as urgent as any that occur in the battlefield**—are constantly taking place in this great industrial district of London. They reach us at the rate of 18 per hour of every working day.

**The sick of the working class** who cannot possibly be nursed in their homes still come to us and cannot be refused.

We are setting aside **20 beds for Wounded Soldiers** without withdrawing any of our beds for our ordinary patients.

**Food, Drugs and Dressings** are all dearer.

All these are reasons why we cannot help asking for additional help, despite all the appeals which are being issued, if we are to do our work properly, and if we are to keep up our 60 years' record of paying our way and never being in debt.

We are appealing to all those who have contributed anything to the Hospital during the last five years to give us extra help in this emergency.

If every subscriber could increase his subscription by 25 per cent. this year we should be free of anxiety.—Yours truly, KNUTSFORD, Chairman; J. G. BROODBANK, Acting Chairman.

---

TO AUTHORS WITH CONFIDENCE.

Kindly send a copy of your latest appeal. We want them from all over the country, please.

